# A Case of Medullary Infarct Causing Central Alveolar Hypoventilation

**DOI:** 10.7759/cureus.17153

**Published:** 2021-08-13

**Authors:** William Lim, May Breitling, Bryan Nugent, Aditi Sinha, Keith Diaz

**Affiliations:** 1 Internal Medicine, Richmond University Medical Center, Staten Island, USA; 2 Pulmonary and Critical Care, Richmond University Medical Center, Staten Island, USA

**Keywords:** ondine's curse, central alveolar hypoventilation, medullary infarct, ischemic cerebrovascular disease, wallenberg syndrome, opalski's syndrome.

## Abstract

Central alveolar hypoventilation (CAH) is a rarely encountered pathology characterized by decreased ventilation due to the loss of autonomic control. Most cases present at birth, as it can be a rare genetic disorder, but we aim to show that it can occur as an acquired condition too. We present a case of a 65-year-old man who developed CAH as a sequela of an ischemic stroke and discuss possible pathophysiology. Increasing awareness and an early detection of this condition can have a significant effect on morbidity and mortality of patients.

## Introduction

Central alveolar hypoventilation (CAH) is characterized by loss of autonomic control of ventilation which can be either due to congenital or acquired causes. This disorder is usually present during the sleep or if severe, may be present even during awake state. Congenital causes are found in children and are caused by a rare genetic disorder because of a defect in the PHOX2B (paired-like homeobox 2B) gene. Acquired causes can be found in adults and are caused by traumatic, ischemic or inflammatory injuries to the brain stem region [1]. We herein present a case of a patient presenting to the emergency department (ED) with left-sided facial numbness, weakness, dysarthria and dysphagia and subsequently developed respiratory failure following an ischemic stroke in the medullary region.

## Case presentation

A 65-year-old man with the past medical history of hypertension, type 2 diabetes mellitus, and prior stroke without residual deficits presented to the emergency department (ED) with left-sided facial numbness, left-sided weakness, dysarthria, and dysphagia for one hour. On arrival to the ED, the patient had a temperature of 98.6 F, blood pressure of 212/102 mmHg, respiratory rate of 14 breaths per minute, and a pulse rate of 66 beats per minute, regular rate and rhythm. A complete blood count (CBC) and basal metabolic panel (BMP) were essentially normal except for high blood glucose levels of 420 mg/dl. Neurological examination revealed equal, round pupils reactive to light, midline tongue, left-sided facial droop, dysphagia and dysarthria. Initial motor power in all four extremities was 5/5. The patient was not given tissue plasminogen activator (TPA) because of accelerated hypertension. 

Initial head computed tomography (CT) and computed tomography angiogram (CTA) of the head and neck showed no acute intracranial abnormalities. Chest X-ray (CXR) showed no acute abnormalities. Aspirin 325 mg and atorvastatin 80 mg were given immediately and aspirin 81 mg daily together with atorvastatin 80 mg bedtime was started. While in the ED, the patient fell while attempting to urinate, sustaining a laceration to the left orbit. Repeat head CT at that time was unchanged. Shortly after, the patient became cyanotic, bradycardic, and unresponsive, requiring intubation with arterial blood gas (ABG) showing pH of 7.21, partial pressure of carbon dioxide (pCO2) of 70 mmHg, partial pressure of oxygen (pO2) of 193 mmHg and bicarbonate (HCO3) of 28 mmHg. Carbon dioxide retention in this patient with no medical history of lung disease indicated possible central respiratory dysfunction. Glasgow coma scale (GCS) at the time of evaluation was 9. Neurological examination, on the next day after intubation, revealed spontaneous movement of right extremities but no spontaneous or pain induced movement of left extremities. The third head CT obtained in light of suspicion of stroke came back negative as well.

**Figure 1:**
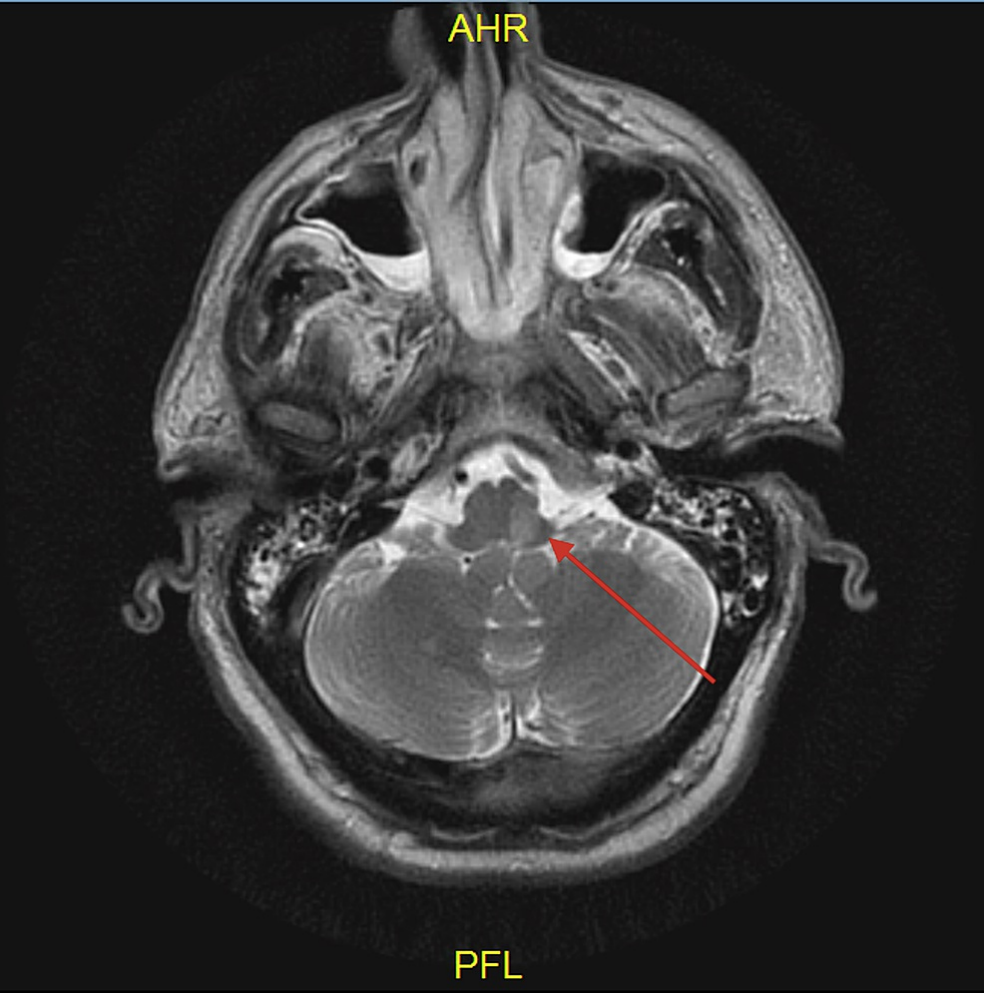
MRI head showing left medullary infarction on axial view. MRI: magnetic resonance imaging.

On day 3, the patient met the weaning parameters on a spontaneous breathing trial and was extubated. But subsequently after extubating, the patient became apneic and required reintubation. Considering that the patient became apneic despite of meeting weaning parameters, suspicion of posterior circulation stroke leading to central alveolar hypoventilation was high. Magnetic resonance imaging (MRI) of the head (Figure 1) was obtained which demonstrated a left medullary infarction. Subsequent attempts to wean off mechanical ventilation failed due to apnea. Patient underwent tracheostomy and remained on mechanical ventilation at discharge.

## Discussion

Central alveolar hypoventilation (CAH), also known as “Ondine’s curse” is a rare condition presenting with respiratory failure during sleep or if severe, even during arousal state [1]. Three major groups of neurons that control the respiration are dorsal respiratory group (the nucleus solitarius) and ventral respiratory group in the medulla (the nucleus ambiguus) and pontine respiratory group (nucleus parabrachialis medialis) [2] and common causes of CAH in adults include brainstem ischemia, mass, infection, demyelinating disease, or anoxic-ischemic damage [3,4]. 

Computed tomography (CT) scan is the preferred imaging study for patients presenting with stroke features because of widespread availability, rapid scan times, and ease of detecting intracranial hemorrhage. However, for diagnosing brain stem ischemic stroke, magnetic resonance imaging (MRI) is the preferred test since brain-stem infarctions are often missed by CT scan because they tend to be small and the resolution of the CT scan in the brain stem is poor [5]. The patient above had 3 consecutive head CTs showing no acute intracranial abnormalities but MRI revealed a left medullary infarction. This case highlights the significance of head MRI in patients with clinical suspicion for a brain-stem stroke. 

In the case presented above, the patient had ipsilateral hemiparesis in addition to the classic symptoms of Wallenberg syndrome caused by lesion in lateral medulla such as ipsilateral facial sensory abnormalities, dysphagia and dysarthria. Wallenberg syndrome is a relatively common posterior circulation cerebrovascular syndrome. However, ipsilateral hemiparesis as part of lateral medullary infarction is otherwise known as Opalski's syndrome. This syndrome is rare, and the functional recovery is not fully understood. Nelles et al reported that patients with lateral medullary infarction have few functional deficits even after completion of rehabilitation [6]. 

Laboratory criteria of CAH are not well-established for diagnosis of CAH [7-9]. The suggested criteria for Ondine’s curse include: (1) hypercapnia and hypoventilation during sleep; (2) normal paO2 when the patient has voluntary breathing while awake and (3) exclusion of other pulmonary diseases and situations which can mimic this disease [10]. But, as mentioned before, there is no absolute agreement on this criteria. Our patient’s clinical presentation and ischemic region in the medullary region supported the diagnosis of central alveolar hypoventilation. 

Respiratory and cardiovascular complications are the main cause of death in patients with lateral medullary infarction [11]. Death related to CAH occurs during sleep, usually due to complete apnea. Pharmacological agents such as acetazolamide, trazodone, acetazolamide, medroxyprogesterone, protriptyline, clomipramine, and caffeine can activate the respiratory center by inducing metabolic acidosis and have been proposed for CAH [12,13]. Supportive measures such as diaphragmatic pacing is also a supportive treatment shown to be effective in CAH [8,14]. But, treatment of CAH is mainly respiratory support with nocturnal assisted ventilation and sometimes tracheostomy, as the cornerstone of treatment [15,16].

## Conclusions

CAH is a rare disorder caused by a variety of mechanisms and should be considered in patients who have deficits in respiratory neural pathways. Prompt recognition of CAH is critical in improving the prognosis of patients. Administering appropriate treatment of assisted ventilation can have a significant impact on a patient’s prognosis.
